# Factors associated with actual mode of delivery among women attending antenatal education at a university hospital in Türkiye: a retrospective observational study

**DOI:** 10.1136/bmjopen-2026-122936

**Published:** 2026-10-22

**Authors:** Cemile Alaca, Menekşe Nazlı Aker, Neslihan Yılmaz Sezer, Satı Tüzün

**Affiliations:** 1 Department of Gynecology and Obstetrics, Ankara University Health Practice and Research Center, Ankara, Turkey; 2 Department of Midwifery, Ankara University Faculty of Nursing, Ankara, Turkey

**Keywords:** OBSTETRICS, Education, Medical, Cesarean Section

## Abstract

**Abstract:**

**Objectives:**

To determine the maternal, obstetric and antenatal education-related factors associated with actual mode of delivery among women attending an open-group antenatal education programme.

**Design:**

Retrospective observational study.

**Setting:**

Antenatal outpatient clinic of a university hospital in Ankara, Türkiye. Data were retrospectively obtained from the hospital information management system and delivery records.

**Participants:**

The source population comprised 1418 women who attended antenatal education between July 2024 and July 2025. Delivery outcome data were available for 641 women, who constituted the analytic sample for mode-of-delivery analyses. As of 31 July 2025, when the data were extracted, 530 women had ongoing pregnancies and 247 had passed their estimated delivery date but had no delivery outcome recorded in the hospital system. Because previous caesarean section resulted in quasicomplete separation, the primary multivariable analysis was restricted to 494 women without a history of previous caesarean section.

**Primary and secondary outcome measures:**

The primary outcome was actual mode of delivery, categorised as vaginal or caesarean delivery. The main explanatory variables were maternal age, parity, gestational week at first educational attendance, pre-education preferred mode of delivery and attendance at each of the four antenatal education sessions. Caesarean indications were additionally summarised descriptively.

**Results:**

Among the 641 women with available delivery outcomes, 390 (60.8%) had a vaginal delivery and 251 (39.2%) underwent caesarean section. In the primary multivariable analysis among women without a previous caesarean section (n=494), increasing maternal age was associated with higher odds of caesarean delivery (adjusted OR (aOR) 1.114, 95% CI 1.056 to 1.176; p<0.001), whereas multiparity was associated with lower odds (aOR 0.117, 95% CI 0.063 to 0.217; p<0.001). An exploratory association was also observed for session 4 attendance (aOR 0.453, 95% CI 0.253 to 0.811; p=0.008). Gestational week at first educational attendance, pre-education preferred mode of delivery and attendance at sessions 1–3 were not independently associated with mode of delivery.

**Conclusions:**

Among women without a history of previous caesarean section, maternal age and parity were independently associated with actual mode of delivery, whereas pre-education delivery preference was not. An exploratory association was observed for session 4 attendance, but this finding should be interpreted cautiously because attendance was non-random and occurred later in pregnancy.

**STRENGTHS AND LIMITATIONS OF THIS STUDY**

- This study examined individual antenatal education sessions, attendance combinations and the gestational timing of each session, allowing a detailed assessment of educational exposure within an open-group programme.
- Routinely collected hospital data were used to evaluate antenatal education attendance and subsequent mode of delivery over a 1-year period.
- The primary multivariable analysis was restricted to women without a history of previous caesarean section and included maternal, obstetric and education-related covariates selected based on clinical relevance, the study objectives and their availability in the routinely collected dataset.
- Potential selection bias related to unavailable delivery outcomes was assessed by comparing women with available and unavailable delivery records and by conducting an inverse-probability-of-selection weighted sensitivity analysis.
- The retrospective observational design, non-random session attendance, incomplete delivery follow-up and residual confounding limit causal interpretation.

## Introduction

 Antenatal education is an important component of prenatal care and aims to provide women and families with knowledge and skills related to pregnancy, childbirth, the postpartum period, breastfeeding and newborn care. Recent systematic reviews indicate that antenatal education may contribute to maternal knowledge, childbirth self-efficacy, psychological preparedness and selected birth outcomes, although considerable heterogeneity exists in programme content, delivery methods and measured outcomes.^1 2^

Childbirth preparation education aims to inform mothers, empower women and support the development of coping skills for the labour process, including pain management, thereby contributing to a more positive childbirth experience. In addition, this education also aims to enhance skills related to infant care, postpartum care, breastfeeding and parenting during the postpartum period.^3 4^ In antenatal education classes in Türkiye, a wide range of topics are covered, including health in pregnancy (anatomy, physiology and the formation of pregnancy), physiological changes during pregnancy, common discomforts and risk conditions, daily life and nutrition during pregnancy, maternal role and communication, preparation for childbirth, pain management during labour, the postpartum period and breastfeeding, newborn care and pregnancy exercises.^5^

Antenatal education may enhance women’s knowledge and informed decision-making regarding self-care, newborn care and postpartum contraceptive methods.^1 2 6^ Several studies have reported that childbirth preparation education is associated with the mode of delivery.^7–9^ However, existing studies have generally evaluated antenatal education as a broad intervention or have focused on the number of sessions attended, while limited evidence is available regarding whether specific educational session content is associated with actual delivery outcomes. To the best of our knowledge, there are no retrospective studies with large sample sizes that examine the relationship between open-group antenatal education and delivery mode in conjunction with specific educational content and obstetric variables. Given the potential short-term maternal risks associated with caesarean section, including infection, haemorrhage and thromboembolic complications, as well as longer-term consequences for subsequent pregnancies and child health, evaluating the factors associated with mode of delivery among women attending antenatal education has become increasingly important.^10^ Accordingly, this study aimed to determine the maternal, obstetric and antenatal education-related factors associated with actual mode of delivery among women attending an open-group antenatal education programme.

## Materials and methods

### Study design

This study was conducted using a retrospective research design.

### Population and sample

The study population consisted of women who received antenatal education at the antenatal outpatient clinic of a university hospital in Ankara. Between July 2024 and July 2025, a total of 1418 women attended the education programme at the clinic. No sampling method was applied, and the entire population was included in the study. Of the 1418 women who attended the antenatal education programme, delivery outcome data were available for 641 women and these participants constituted the analytic sample for analyses of actual mode of delivery. As of 31 July 2025, when the data were extracted, 530 women had ongoing pregnancies and had therefore not yet reached the delivery outcome window. Among the remaining women, 247 had passed their estimated date of delivery but no delivery outcome was available in the hospital records. Women with ongoing pregnancies were not considered to have missing delivery outcomes. A participant flow diagram was constructed to show the follow-up status of the full cohort.

At the hospital, antenatal education is provided to pregnant women in the outpatient waiting area; therefore, it is conducted as an open-group format. Each session lasts approximately 40–50 min. All sessions were delivered by the same educator at a fixed time each day in the outpatient waiting area and the programme structure and session content remained consistent throughout the study period. No predefined class groups were formed; participation depended on which pregnant women were present in the outpatient waiting area at the scheduled education time. The antenatal education programme comprised four predefined session topics. Sessions were not assigned according to gestational age and did not follow a fixed calendar rotation. At the scheduled education time, pregnant women present in the outpatient waiting area were offered the available session topics, and the topic delivered that day was selected according to the preferences of the women present. There were no predefined gestational-age eligibility windows for any of the four sessions. Therefore, the observed gestational timing of attendance reflects actual attendance patterns rather than programme-defined gestational-age requirements. Because of the open-group structure, women could attend one or more sessions depending on the timing of their antenatal visit and availability; therefore, attendance at each session was evaluated separately. The first session included an introduction and hospital orientation, male and female reproductive anatomy and physiology, the formation of pregnancy, physical and psychological changes during pregnancy, fetal development and a question-and-answer discussion. The second session covered daily life during pregnancy, nutrition, exercise, sexual life, medication use, antenatal follow-up, routine tests, immunisation and common problems with their management, followed by a discussion. The third session addressed danger signs during pregnancy and appropriate responses, the labour process, hospital procedures, preparation for childbirth, including a hospital bag, modes of delivery, breathing exercises and pain management, procedures during labour, umbilical cord blood donation and discussion. The fourth session focused on breastfeeding and breast milk, infant care, newborn screening and immunisation, postpartum care, postpartum nutrition and exercise, postpartum sexual life and contraception, as well as hospital visits and emergency situations for mother and newborn, followed by a discussion.

### Data collection

Data were retrospectively obtained from the hospital information management system and delivery records. The following information was recorded for each participant: age, parity, gestational week at the time of education, participation in antenatal education sessions, preferred mode of delivery prior to education, previous caesarean section history and the actual mode of delivery. Parity status was categorised as nullipara or multipara. Participation in each antenatal education session was coded separately as attended or not attended. Preferred mode of delivery was recorded before antenatal education and categorised according to the available records. The actual mode of delivery was categorised as vaginal delivery or caesarean section.

### Patient and public involvement

None.

### Data analysis

Data were analysed using IBM SPSS Statistics V.27.0. Descriptive statistics were presented as n (%), mean±SD, or median (25th–75th percentile), as appropriate. Pearson’s χ^2^ test was used to examine associations between categorical variables and the actual mode of delivery. Session-attendance combinations, pairwise associations between session attendance indicators (using phi coefficients) and gestational timing of attendance at each session were also summarised.

Because a history of previous caesarean section resulted in quasicomplete separation, the primary multivariable analysis was restricted to women without a history of previous caesarean section (n=494). Binary logistic regression was performed with the actual mode of delivery coded as 0=vaginal delivery and 1=caesarean delivery. The model included maternal age, parity, gestational week at first educational attendance, pre-education preferred mode of delivery and attendance at sessions 1–4. Covariates were selected based on their clinical relevance to mode of delivery, the study objectives, and their availability in the routinely collected dataset. Linearity of the continuous predictors with the logit was assessed using the Box-Tidwell approach, with no evidence of violation for maternal age or gestational week at first educational attendance. Adjusted ORs (aORs) with 95% CIs were reported. Model fit was assessed using the Hosmer-Lemeshow test and Nagelkerke R^2^.

To assess potential selection bias related to delivery outcome availability, baseline and education-related characteristics were compared between women with available delivery outcomes (n=641) and women whose estimated date of delivery had passed but whose delivery outcome was unavailable (n=247). Women with ongoing pregnancies at the data extraction date were excluded from this comparison because their delivery outcomes had not yet occurred. Age was compared using Welch’s independent-samples t-test, gestational week at first educational attendance and number of sessions attended using the Mann-Whitney U test, and categorical variables using Pearson’s χ^2^ test. The results are presented in online supplemental table 1. As a sensitivity analysis to address potential selection related to delivery-outcome availability, stabilised inverse-probability-of-selection weights were estimated among women without a history of previous caesarean section who had reached the delivery-outcome window. This population comprised 494 women with observed delivery outcomes and 217 women whose delivery outcomes were unavailable. The probability of outcome availability was estimated using logistic regression including maternal age, parity, gestational week at first educational attendance, pre-education preferred mode of delivery and attendance at sessions 1–4. Stabilised weights were calculated and applied to the 494 women with observed delivery outcomes, and the primary multivariable logistic regression model was repeated as a weighted sensitivity analysis. A p value of <0.05 was considered statistically significant. This study was reported in accordance with the Strengthening the Reporting of Observational Studies in Epidemiology (STROBE) guidelines and the REporting of studies Conducted using Observational Routinely-collected health Data (RECORD) extension.

## Results

Delivery outcome data were available for 641 women, who constituted the analytic sample for mode-of-delivery analyses (figure 1). Among the 641 women included in the analytic sample, the mean age was 29.00±5.13 years, and the median gestational week at first educational attendance was 24 (Q1–Q3: 17–33). The median number of educational sessions attended was 1 (Q1–Q3: 1–2); 60.1% of women attended one session, 28.5% attended two sessions, 10.5% attended three sessions and 0.9% attended all four sessions. Attendance rates for sessions 1, 2, 3 and 4 were 24.6%, 37.9%, 43.4% and 46.3%, respectively. Before education, 72.1% preferred vaginal delivery, 25.0% preferred caesarean delivery and 3.0% were undecided. Of the analytic sample, 37.8% were nulliparous, 22.9% had a previous caesarean section and 60.8% ultimately had a vaginal delivery (table 1).

**Figure 1:**
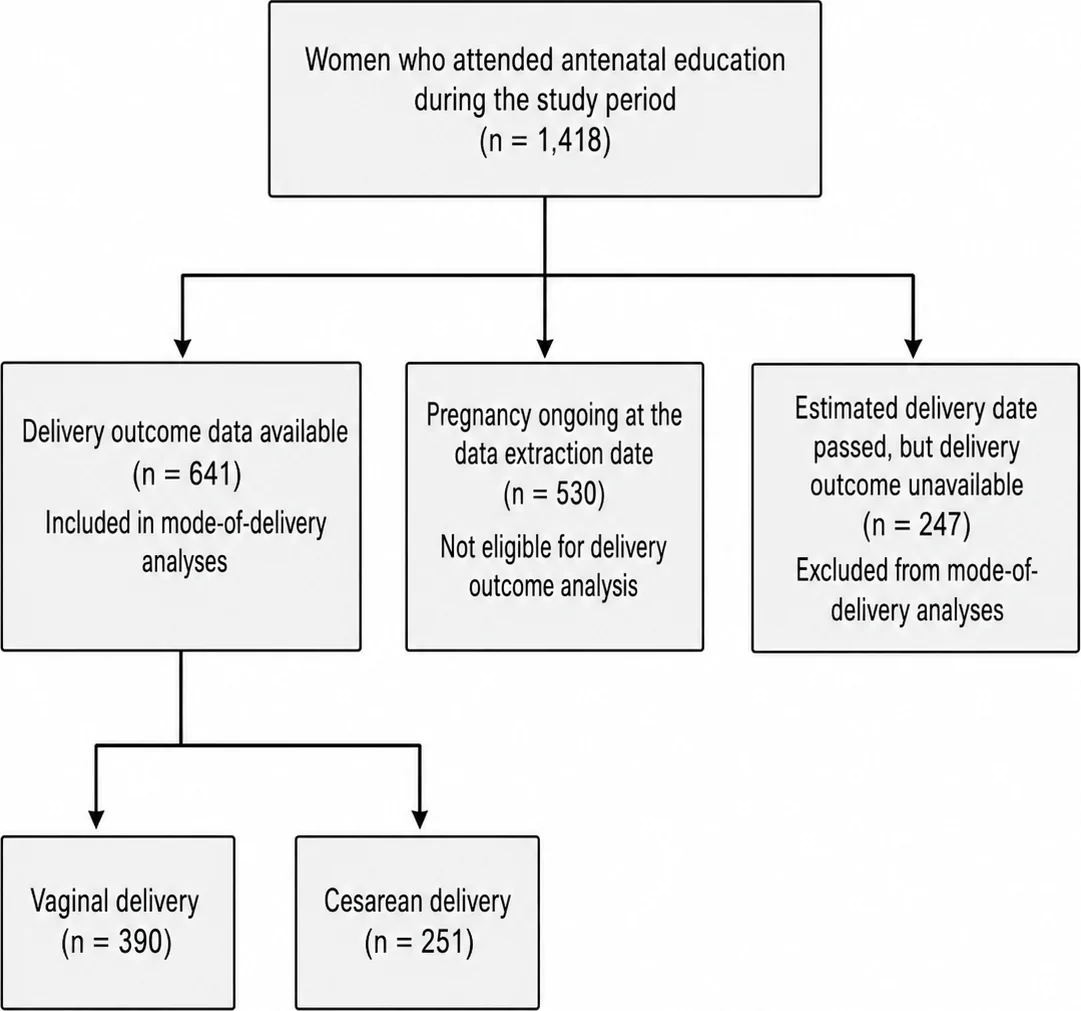
Participant flow diagram.

**Table 1:** Baseline, education-related and obstetric characteristics of the analytic sample (n=641)

Characteristics	n (%)/summary statistic
Age, years, mean±SD	29.00±5.13
Gestational week at first educational attendance, median (Q1–Q3)	24 (17–33)
Number of educational sessions attended, median (Q1–Q3)	1 (1–2)
One session	385 (60.1)
Two sessions	183 (28.5)
Three sessions	67 (10.5)
Four sessions	6 (0.9)
Educational session attendance	
Session 1	158 (24.6)
Session 2	243 (37.9)
Session 3	278 (43.4)
Session 4	297 (46.3)
Pre-education preferred mode of delivery	
Vaginal delivery	462 (72.1)
Caesarean delivery	160 (25.0)
Undecided	19 (3.0)
Parity	
Nullipara	242 (37.8)
Multipara	399 (62.2)
Previous caesarean section	
No	494 (77.1)
Yes	147 (22.9)
Actual mode of delivery	
Vaginal delivery	390 (60.8)
Caesarean delivery	251 (39.2)

Data are presented as n (%), mean±SD, or median (25th–75th percentile), as appropriate.

Compared with women whose estimated delivery date had passed but whose delivery outcome was unavailable (n=247), women in the analytic sample (n=641) had a slightly higher gestational age at first educational attendance (median 24 (17–33) vs 23 (19–30) weeks; p=0.029) and attended more educational sessions (median 1 (1–2) vs 1 (1–1); p<0.001). Attendance at session 3 (43.4% vs 32.4%; p=0.003) and session 4 (46.3% vs 31.6%; p<0.001), multiparity (62.2% vs 49.4%; p<0.001) and previous caesarean history (22.9% vs 12.1%; p<0.001) were also more frequent in the analytic sample. No differences were observed in age (p=0.961), pre-education delivery preference (p=0.332) or attendance at session 1 (p=0.165) or session 2 (p=0.685) (online supplemental table 1). A wide range of session-attendance combinations was observed, reflecting the open-group structure of the education programme. Single-session attendance was most common (60.1%), particularly attendance at session 4 only (25.4%), session 3 only (17.2%) and session 2 only (11.7%). Only six women (0.9%) attended all four sessions. All observed attendance combinations are presented in online supplemental table 2. Pairwise associations among session attendance indicators were generally weak. Session 1 attendance was positively associated with session 2 attendance (phi=0.128, p=0.001), whereas negative associations were observed between sessions 1 and 4 (phi=−0.227, p<0.001), sessions 2 and 4 (phi=−0.281, p<0.001) and sessions 3 and 4 (phi=−0.314, p<0.001). The association between sessions 1 and 3 was not statistically significant (phi=−0.055, p=0.164), while the association between sessions 2 and 3 was weak (phi=−0.087, p=0.028) (online supplemental table 3). The median gestational week at attendance was 14 (13–16) weeks for session 1, 22 (20–23) weeks for session 2, 28 (26–31) weeks for session 3, and 36 (33–38) weeks for session 4 (online supplemental table 4).

The proportions of vaginal and caesarean delivery did not differ according to attendance at sessions 1, 2 or 3 (p>0.05). Among women who attended session 4, 67.7% had a vaginal delivery and 32.3% had a caesarean delivery, compared with 54.9% and 45.1%, respectively, among women who did not attend session 4 (χ²=10.850; p<0.001) (table 2). In the overall analytic sample (n=641), when the mode of delivery was compared according to parity, the caesarean delivery rate was significantly higher among multiparous women than among nulliparous women (42.4% vs 33.9%; χ²=4.538; p=0.033).

**Table 2:** Actual mode of delivery according to antenatal education session attendance in the analytic sample (n=641)

Session	Attendance	Vaginal delivery, n (%)	Caesarean delivery, n (%)	χ^2^	P value
Session 1	Did not attend	297 (61.5)	186 (38.5)	0.346	0.557
	Attended	93 (58.9)	65 (41.1)		
Session 2	Did not attend	247 (62.1)	151 (37.9)	0.654	0.419
	Attended	143 (58.8)	100 (41.2)		
Session 3	Did not attend	225 (62.0)	138 (38.0)	0.457	0.499
	Attended	165 (59.4)	113 (40.6)		
Session 4	Did not attend	189 (54.9)	155 (45.1)	10.850	<0.001
	Attended	201 (67.7)	96 (32.3)		

Percentages are calculated within each session-attendance group. Pearson’s χ2 test was used to compare the distribution of actual mode of delivery between women who attended and did not attend each session.

Among women without a history of previous caesarean section, the proportions of vaginal and caesarean delivery did not differ according to attendance at sessions 1, 2 or 3 (p>0.05). Among women who attended session 4, 83.7% had a vaginal delivery and 16.3% had a caesarean delivery, compared with 73.7% and 26.3%, respectively, among those who did not attend session 4 (χ²=7.257; p=0.007) (table 3).

**Table 3:** Actual mode of delivery according to antenatal education session attendance among women without a history of previous caesarean section (n=494)

Session	Attendance	Vaginal delivery, n (%)	Caesarean delivery, n (%)	χ^2^	P value
Session 1	Did not attend	296 (78.1)	83 (21.9)	0.189	0.664
	Attended	92 (80.0)	23 (20.0)		
Session 2	Did not attend	245 (78.8)	66 (21.2)	0.028	0.868
	Attended	143 (78.1)	40 (21.9)		
Session 3	Did not attend	223 (79.1)	59 (20.9)	0.112	0.738
	Attended	165 (77.8)	47 (22.2)		
Session 4	Did not attend	188 (73.7)	67 (26.3)	7.257	0.007
	Attended	200 (83.7)	39 (16.3)		

Percentages are calculated within each session-attendance group. Pearson’s χ2 test was used to compare the distribution of actual mode of delivery between women who attended and did not attend each session.

Among the 251 women who underwent caesarean delivery, the most common indication was repeat caesarean section (57.8%), followed by fetal distress (15.5%), malpresentation (6.8%) and cephalopelvic disproportion (4.8%). Among women without a history of previous caesarean section who underwent caesarean delivery (n=106), fetal distress was the most frequent indication (36.8%), followed by malpresentation (16.0%), cephalopelvic disproportion (11.3%), maternal request (9.4%) and failed induction of labour for post-term pregnancy (8.5%) (online supplemental table 5).

In the multivariable logistic regression analysis restricted to women without a history of previous caesarean section (n=494), the overall model was statistically significant (χ²(9)=71.795; p<0.001), with a Nagelkerke R² of 0.209. The Hosmer-Lemeshow test was χ²=5.603 (df=8; p=0.692). Increasing maternal age was associated with higher odds of caesarean delivery (aOR=1.114; 95% CI 1.056 to 1.176; p<0.001), whereas multiparity was associated with lower odds of caesarean delivery (aOR=0.117; 95% CI 0.063 to 0.217; p<0.001). An exploratory association was also observed for session 4 attendance (aOR=0.453; 95% CI 0.253 to 0.811; p=0.008). Gestational week at first educational attendance, preferred mode of delivery and attendance at sessions 1–3 were not independently associated with mode of delivery (p>0.05) (table 4).

**Table 4:** Multivariable logistic regression analysis of factors associated with caesarean delivery among women without a history of previous caesarean section (n=494)

Variable	B	SE	Wald	P value	Adjusted OR	95% CI
Age, years	0.108	0.027	15.594	<0.001	1.114	1.056 to 1.176
Gestational week at first educational attendance	−0.006	0.025	0.052	0.820	0.994	0.946 to 1.045
Multiparous	−2.147	0.317	45.956	<0.001	0.117	0.063 to 0.217
Preferred caesarean delivery	0.426	0.528	0.652	0.419	1.532	0.544 to 4.312
Undecided regarding preferred delivery mode	−0.164	0.572	0.082	0.775	0.849	0.277 to 2.605
Session 1 attendance	−0.186	0.428	0.189	0.664	0.830	0.359 to 1.920
Session 2 attendance	−0.245	0.343	0.511	0.475	0.783	0.400 to 1.532
Session 3 attendance	−0.295	0.290	1.032	0.310	0.745	0.422 to 1.315
Session 4 attendance	−0.791	0.297	7.115	0.008	0.453	0.253 to 0.811

Model χ2(9)=71.795, p<0.001; Nagelkerke R2=0.209; Hosmer-Lemeshow p=0.692. The dependent variable was coded as 0=vaginal delivery and 1=caesarean delivery. Reference categories were nulliparous, preferred vaginal delivery and non-attendance at each education session.

In the inverse-probability-of-selection weighted sensitivity analysis, the estimates were broadly consistent with those of the primary analysis. Maternal age remained associated with higher odds of caesarean delivery (aOR=1.097; 95% CI 1.041 to 1.156; p<0.001), whereas multiparity remained associated with lower odds of caesarean delivery (aOR=0.139; 95% CI 0.076 to 0.255; p<0.001). Session 4 attendance was also associated with lower odds of caesarean delivery in the weighted analysis (aOR=0.510; 95% CI 0.285 to 0.913; p=0.023). Gestational week at first educational attendance, pre-education preferred mode of delivery and attendance at sessions 1–3 remained non-significant (online supplemental table 6).

## Discussion

This study examined factors associated with actual mode of delivery among women attending antenatal education. Among women without a history of previous caesarean section, maternal age and parity were independently associated with mode of delivery, whereas pre-education delivery preference was not. An exploratory association was also observed for session 4 attendance, but this finding requires cautious interpretation because of non-random attendance and its later observed timing in pregnancy.

In Türkiye, the Ministry of Health recommends preventing unnecessary caesarean sections and promoting vaginal delivery unless medically indicated.^11^ In the present study, the caesarean section rate was 39.2%, which is lower than the national rate of 61.2% reported in 2024. However, the WHO, based on systematic reviews and analyses conducted in 2015, has reported that caesarean section rates above 10–15% are not associated with reductions in maternal and neonatal mortality.^12^ In addition, the European Association of Perinatal Medicine and the European Midwives Association recommend that national caesarean section rates should be between 15% and 20%.^13^ The rate observed in this study remains considerably higher than these recommended levels. Although caesarean section is a life-saving intervention when medically indicated, it is also associated with short-term and long-term adverse health outcomes for both women and children.^10^ Therefore, it is important to continue developing and implementing evidence-based strategies aimed at reducing unnecessary caesarean sections.

In this study, maternal age was identified as an independent factor associated with higher odds of caesarean delivery. Each 1-year increase in maternal age was associated with an approximately 11% increase in the adjusted odds of caesarean delivery. Consistent with our findings, the literature widely reports that advanced maternal age is associated with higher rates of caesarean delivery.^14–16^ This finding may be related to the higher frequency of obstetric complications and interventions observed with advancing maternal age. Previous studies have similarly shown that advanced maternal age is associated with an increased risk of adverse pregnancy outcomes, including caesarean delivery.^17^

Parity was also independently associated with mode of delivery. Among women without a history of previous caesarean section, multiparous women had substantially lower adjusted odds of caesarean delivery than nulliparous women (aOR=0.117; 95% CI 0.063 to 0.217). This finding is consistent with previous studies indicating that nulliparity is associated with a higher risk of caesarean delivery, whereas multiparity is generally associated with a greater likelihood of vaginal birth.^18^ In the overall analytic sample, however, the crude caesarean rate was higher among multiparous women. This difference may be explained by the higher prevalence of previous caesarean history among multiparous women and supports the decision to restrict the primary multivariable analysis to women without a previous caesarean section. The association observed in the present study supports parity as an important obstetric factor related to mode of delivery among women without a previous caesarean section.

Another notable finding of this study was that the preferred mode of delivery before education was not significantly associated with the actual mode of delivery. This suggests that maternal preferences alone may not be sufficient to predict delivery outcomes and that the mode of delivery is shaped by a combination of obstetric factors and educational exposure. In this study, the preferred mode of delivery was assessed only before the education, whereas post-education delivery preference was not assessed and the actual mode of delivery was evaluated as the outcome variable. Therefore, the lack of a statistically significant association between pre-education preferences and delivery outcomes should not be interpreted as direct evidence of the effect of antenatal education. Instead, it may indicate that pre-education preference alone was insufficient to predict the actual mode of delivery after obstetric and educational variables were considered. Consistent with this interpretation, the literature indicates that antenatal education is associated with women’s delivery preferences and delivery outcomes. Previous studies suggest that such education may increase women’s knowledge, reduce fear and anxiety related to childbirth and promote more positive attitudes toward vaginal delivery.^1 3 8 9^

An exploratory association between session 4 attendance and lower odds of caesarean delivery was observed in the primary multivariable analysis and remained statistically significant in the weighted sensitivity analysis. This association was also observed in the inverse-probability-of-selection weighted sensitivity analysis, in which the magnitude of the association was attenuated but remained statistically significant. However, this finding requires cautious interpretation. Although none of the sessions were assigned according to gestational age and there were no predefined gestational-age eligibility windows, session 4 was observed to be attended later in pregnancy, at a median gestational age of 36 weeks. Because gestational age at delivery was not available, it was not possible to determine whether some women classified as non-attenders had delivered before having an opportunity to attend session 4. Accordingly, the observed association may reflect differences in opportunity for attendance, characteristics of women who remained pregnant and continued attending antenatal care later in gestation, or other unmeasured clinical factors, rather than an effect attributable to the content of session 4 itself. The session 4 finding should therefore be regarded as exploratory and hypothesis-generating and requires confirmation in prospective studies that explicitly account for eligibility and timing of attendance.

### Limitations

Delivery outcomes were unavailable for some women whose estimated delivery date had passed, and differences in education attendance and parity indicated the potential for selection bias. Although an inverse-probability-of-selection weighted sensitivity analysis produced results broadly consistent with the primary analysis, residual selection bias due to unmeasured factors cannot be excluded. The retrospective observational design and non-random attendance within the open-group education programme also limit causal interpretation, particularly for session 4, which was generally attended later in pregnancy. Although clinical indications for caesarean delivery were available and are reported descriptively, these indications were not available as comparable pre-delivery characteristics for all women in the analytic sample and therefore could not be incorporated as covariates in the mode-of-delivery model. Because four session-specific associations were examined without formal adjustment for multiple comparisons, the session 4 finding should be considered exploratory and warrants confirmation in prospective studies. Finally, delivery preference was assessed only before education, and posteducation changes in preference could not be evaluated. As this was a single-centre study conducted within an open-group antenatal education programme at a university hospital, the findings may not be directly generalisable to settings with different antenatal education structures, populations or referral patterns.

## Conclusion

Considering the increasing rates of caesarean section, it is important for nurses and midwives involved in antenatal care to recognise the factors associated with mode of delivery and to support evidence-based approaches to childbirth preparation. In this study, among women without a history of previous caesarean section, maternal age and parity were independently associated with actual mode of delivery, whereas pre-education delivery preference was not. An exploratory association was also observed for session 4 attendance; however, because session 4 was attended later in pregnancy and attendance was non-random, this finding requires confirmation in prospective studies that explicitly account for eligibility and timing of attendance.

## Supplementary material

10.1136/bmjopen-2026-122936 online supplemental file 1

## Data Availability

Data are available upon reasonable request.
