# Supplementary material for: Factors associated with actual mode of delivery among women attending antenatal education at a university hospital in Türkiye: a retrospective observational study
Source: BMJ Open. 2026 Oct 22;16(10):e122936. doi: 10.1136/bmjopen-2026-122936 (PMC13691776; doi:10.1136/bmjopen-2026-122936)
Supplement: Supplementary file 1 [file bmjopen-16-10-s001.pdf]

Supplementary Table 1. Comparison of baseline and education-related characteristics according to delivery outcome availability

Variable	Delivery outcome available n=641	Delivery outcome unavailable n=247	p
Age, mean ± SD	29.00 ± 5.13	29.02 ± 5.82	0.961
Gestational week at first education, median (Q1–Q3)	24 (17–33)	23 (19–30)	0.029
Number of sessions attended, median (Q1–Q3)	1 (1–2)	1 (1–1)	<0.001
Session 1 attendance, n (%)	158 (24.6)	50 (20.2)	0.165
Session 2 attendance, n (%)	243 (37.9)	90 (36.4)	0.685
Session 3 attendance, n (%)	278 (43.4)	80 (32.4)	0.003
Session 4 attendance, n (%)	297 (46.3)	78 (31.6)	<0.001
Preferred vaginal delivery, n (%)	462 (72.1)	189 (76.5)	
Preferred cesarean delivery, n (%)	160 (25.0)	50 (20.2)	0.332
Undecided, n (%)	19 (3.0)	8 (3.2)	
Multipara, n (%)	399 (62.2)	122 (49.4)	<0.001
Previous cesarean, n (%)	147 (22.9)	30 (12.1)	<0.001

Note: Data are presented as mean ± standard deviation, median (25th–75th percentile), or n (%), as appropriate. Comparisons were performed using Welch's independent-samples t-test, the Mann–Whitney U test, or Pearson's chi-square test.

Supplementary Table 2. Observed combinations of antenatal education session attendance in the analytic sample (n=641)

Session attendance combination	n (%)
Session 1 only	37 (5.8)
Session 2 only	75 (11.7)
Session 3 only	110 (17.2)
Session 4 only	163 (25.4)
Sessions 1 + 2	33 (5.1)
Sessions 1 + 3	23 (3.6)
Sessions 1 + 4	12 (1.9)
Sessions 2 + 3	43 (6.7)
Sessions 2 + 4	28 (4.4)
Sessions 3 + 4	44 (6.9)
Sessions 1 + 2 + 3	23 (3.6)
Sessions 1 + 2 + 4	15 (2.3)
Sessions 1 + 3 + 4	9 (1.4)
Sessions 2 + 3 + 4	20 (3.1)
Sessions 1 + 2 + 3 + 4	6 (0.9)

Supplementary Table 3. Pairwise associations among antenatal education session attendance indicators (n=641)

Session pair	χ^2	p	Phi
Session 1–2	10.438	.001	0.128
Session 1–3	1.936	.164	-0.055
Session 1–4	32.898	<.001	-0.227
Session 2–3	4.837	.028	-0.087
Session 2–4	50.649	<.001	-0.281
Session 3–4	63.374	<.001	-0.314

Note: Pearson's chi-square test was used to assess pairwise associations between session attendance indicators. Phi coefficients are reported as measures of association; negative values indicate inverse associations between attendance at the paired sessions.

Supplementary Table 4. Gestational timing of attendance at each antenatal education session in the analytic sample (n=641)

Session	n	Gestational week, median (Q1–Q3)	Min–max
Session 1	158	14 (13–16)	12–35
Session 2	243	22 (20–23)	16–40
Session 3	278	28 (26–31)	12–40
Session 4	297	36 (33–38)	12–40

Note: Data are presented as median (25th–75th percentile) and minimum–maximum gestational weeks among women who attended each respective session.

Supplementary Table 5. Indications for cesarean delivery in the analytic sample

Cesarean indication	All cesarean deliveries (n=251), n (%)	Cesarean deliveries among women without previous cesarean section (n=106), n (%)
Repeat cesarean section	145 (57.8)	—
Fetal distress	39 (15.5)	39 (36.8)
Malpresentation	17 (6.8)	17 (16.0)
Multiple pregnancy	3 (1.2)	3 (2.8)
Maternal request	10 (4.0)	10 (9.4)
Maternal disease	8 (3.2)	8 (7.6)
Premature rupture of membranes	1 (0.4)	1 (0.9)
Cord prolapse	1 (0.4)	1 (0.9)
Failed induction of labour for post-term pregnancy	9 (3.6)	9 (8.5)
Cephalopelvic disproportion	12 (4.8)	12 (11.3)
Placenta previa	4 (1.6)	4 (3.8)
Oligohydramnios	2 (0.7)	2 (2.0)
**Total**	**251 (100.0)**	**106 (100.0)**

Note: Percentages are calculated within each column.

Supplementary Table 6. Primary and inverse-probability-of-selection weighted multivariable analyses among women without a history of previous cesarean section

Variable	Primary analysis aOR (95% CI)	p	IPSW sensitivity analysis aOR (95% CI)	p
Age, years	1.114 (1.056–1.176)	<0.001	1.097 (1.041–1.156)	<0.001
Gestational week at first educational attendance	0.994 (0.946–1.045)	0.820	0.998 (0.952–1.045)	0.923
Multiparous	0.117 (0.063–0.217)	<0.001	0.139 (0.076–0.255)	<0.001
Preferred cesarean delivery	1.532 (0.544–4.312)	0.419	1.605 (0.646–3.992)	0.309
Undecided regarding preferred delivery mode	0.849 (0.277–2.605)	0.775	1.087 (0.371–3.182)	0.879
Session 1 attendance	0.830 (0.359–1.920)	0.664	0.783 (0.344–1.781)	0.559
Session 2 attendance	0.783 (0.400–1.532)	0.475	0.749 (0.390–1.438)	0.385
Session 3 attendance	0.745 (0.422–1.315)	0.310	0.827 (0.476–1.440)	0.503
Session 4 attendance	0.453 (0.253–0.811)	0.008	0.510 (0.285–0.913)	0.023

Note: Both models were restricted to women without a history of previous cesarean section and with observed delivery outcomes (n=494). For the sensitivity analysis, stabilized inverse-probability-of-selection weights were estimated using a selection model fitted among 711 women who had reached the delivery-outcome window and had no history of previous cesarean section (494 with observed outcomes and 217 with unavailable outcomes). Reference categories were nulliparous, preferred vaginal delivery, and non-attendance at each education session. aOR, adjusted odds ratio; CI, confidence interval; IPSW, inverse-probability-of-selection weighting.
