# Supplementary material for: Factors associated with actual mode of delivery among women attending antenatal education at a university hospital in Türkiye: a retrospective observational study
Source: BMJ Open. 2026 Oct 22;16(10):e122936. doi: 10.1136/bmjopen-2026-122936 (PMC13691776; doi:10.1136/bmjopen-2026-122936)
Supplement: Supplementary file 2 [file bmjopen-2026-122936.reviewer_comments.pdf]

PEER REVIEW HISTORY

BMJ Open publishes all reviews undertaken for accepted manuscripts. Reviewers are asked to complete a checklist review form and are provided with free text boxes to elaborate on their assessment. These free text comments are reproduced below.

ARTICLE DETAILS

Title (Provisional)

Factors Associated with Actual Mode of Delivery Among Women Attending Antenatal Education at a University Hospital in Türkiye: A Retrospective Observational Study

Authors

Alaca, Cemile; Aker, Menekşe; Yılmaz Sezer, Neslihan; Tüzün, Satı

VERSION 1 - REVIEW

**Reviewer**	**1**
**Name**	**Kara, Pınar**
**Affiliation**	**Kahramanmaraş İstiklal University, Nursing Department**
**Date**	**01-Jul-2026**
**COI**	**Nine**

Dear Authors,

I have reviewed your study examining the relationship between prenatal education processes, birth preferences, and final delivery methods of pregnant women with great interest and satisfaction. I congratulate you on this valuable work, which has the potential to make a significant contribution to the maternal health literature, both clinically and academically.

The use of logistic regression modeling in analyzing multifactorial dynamics such as birth preferences and actual delivery methods has significantly elevated the level of scientific evidence in the study. The presentation of the tabular data is extremely transparent and systematically structured. The exceptional meticulousness with which all statistical details are interpreted in depth, guiding the reader without getting lost in the details, has increased the readability of the study. The findings have been rationalized within a framework of strong scientific foundations, blended with current and high-quality literature.

To further enhance the quality of your study and strengthen its visibility in international indexes, I am submitting a few minor points that I suggest you review in the main text, along with the attached files.

Sincerely,

Referee recommendations

"What this study adds

This study found that attendance at the postpartum-focused fourth antenatal education session was associated with a lower likelihood of cesarean delivery, while pre-education delivery preference was not independently associated with actual mode of delivery."

Recommendation: Reducing the sentence to a few short sentences will make it easier to understand, thus improving the fluency of the article.

"Given the short- and long-term adverse health effects of cesarean section for both women and children[11],….."

Recommendation: Sharing short-term and long-term results, prioritizing those that are most valuable, can further enhance the overall picture.

"The analysis indicated that increasing age (OR=1.121; $p<0.001$) and a history of previous cesarean section (OR=821.402; $p<0.001$) were significantly associated with an increased likelihood of cesarean delivery. In contrast, multiparity (OR=0.114; $p<0.001$) and attendance at the fourth education session (OR=0.482; $p=0.011$) were significantly associated with a decreased likelihood of cesarean delivery."

Recommendation: Since the table presents data on the number of pregnancies, it would be appropriate to make a comment related to pregnancies here as well. If a comment regarding parity is desired, adding this data to the table would support a clearer report.

"This finding may be explained by the increase in obstetric risks with advancing maternal age, as well as clinicians' tendency to prefer cesarean delivery as a….."

Recommendation: In descriptive/cross-sectional studies, it is flawed to conclude the discussion by establishing cause-and-effect relationships. Rather, it is recommended that the discussion be concluded with suggestions/inferences aimed at reducing the tendency for cesarean sections in women of this age or encouraging vaginal delivery. This could include suggesting ways to lower the age of childbirth.

"The extremely high likelihood of repeat cesarean delivery among women with a prior cesarean section (OR=821.402) is consistent with the literature."

Recommendation: It is recommended to include supporting literature evidence.

"This statistical phenomenon reflects the clinical reality of the "once a cesarean, always a cesarean" approach and the low rates of vaginal birth after cesarean (VBAC)[19-21]. Therefore, this finding should be interpreted not only as a clinical indicator of repeat cesarean tendency, but also as a statistical limitation related to model stability."

Recommendation: It would be better to update the statement as an inference sentence.

CONCLUSION

Recommendation: Before we go any further, it would be helpful to include the strengths and limitations of the study.

**** **The reviewer provided a marked copy with additional comments. Please contact the publisher for full details.** ****

**Reviewer**	**2**
**Name**	**Lamadrid-Figueroa, Hector**
**Affiliation**	**National Institute of Public Health, Department of Perinatal Health**
**Date**	**22-Jul-2026**
**COI**	**None**

Statistical review

This review is restricted to the statistical methods, analysis, reporting, and interpretation.

The study addresses a relevant question and includes a reasonably large initial cohort. However, several important statistical issues currently limit interpretation of the reported association between attendance at the fourth educational session and mode of delivery.

Major comments

1. Selection into the analytic sample

Delivery outcomes were available for only 641 of 1,418 women: 45.2% of the initial cohort. Thus, 777 women (54.8%) were excluded from all outcome analyses because they delivered elsewhere or their records were unavailable. This cannot be treated as ordinary missingness without further investigation. Choice of delivery hospital may be related to educational attendance, pregnancy risk, planned cesarean delivery, socioeconomic circumstances, or other determinants of delivery mode.

The authors should:

• provide a participant flow diagram and detailed reasons for outcome non-availability;

• compare the 641 included women with the 777 excluded women on every available baseline and attendance variable;

• present Table 1 separately for the analytic sample, rather than combining characteristics of all 1,418 women with outcomes from only 641;

• discuss the likely direction and consequences of selection bias; and

• consider an inverse-probability-of-selection weighted sensitivity analysis using variables available for the complete cohort, or other methods for controlling selection bias.

Even after such an analysis, the target population should be described as women attending antenatal education who subsequently delivered at the same hospital, rather than all attendees.

2. Attendance at the fourth session cannot presently be interpreted as an effect of its content

Attendance was neither randomized nor assigned according to session content. Because women could attend different and overlapping combinations of sessions, fourth-session attendance may be a marker of earlier entry into antenatal care, greater healthcare engagement, more visits, scheduling opportunity, socioeconomic characteristics, or differences in pregnancy risk. Adjustment for "gestational week at education" alone is unlikely to address this.

The authors should report:

• the distribution of the number of sessions attended in the 641-woman analytic sample;

• all observed attendance combinations and the correlations among the four attendance indicators;

• the exact scheduling and sequence of sessions;

• gestational age at each attended session, not merely an ambiguously defined gestational week;

• whether educators, calendar periods, or class groups differed; and

• an a priori rationale for confounder selection.

Clinically important confounders appear to be unavailable or omitted, including indications for cesarean delivery, fetal presentation, pregnancy complications, induction or onset of labor, previous vaginal birth, planned cesarean delivery, antenatal-care utilization, and relevant socioeconomic factors. Without adequate control for these variables, the analysis cannot support conclusions about the distinctive value of postpartum-focused content.

3. Quasi-complete separation is acknowledged but not addressed analytically

From Tables 1 and 3, it can be inferred that 145 of 147 women with a previous cesarean underwent another cesarean, while only two delivered vaginally. Consequently, the maximum-likelihood estimate for previous cesarean is severely affected by quasi-complete separation, producing the unstable OR of 821.4.

Simply acknowledging this limitation in the Discussion does not resolve the problem. The authors should either:

• make the adjusted analysis among women without a previous cesarean the primary analysis for the educational-session question; or

• refit the full model using a separation-robust method, such as Firth penalized logistic regression or an appropriately specified Bayesian model.

Table 3 contains only unadjusted chi-square analyses. An adjusted model among the 494 women without previous cesarean is essential. An interaction between previous cesarean and session attendance could theoretically be examined, although only two vaginal births among women with previous cesarean make meaningful estimation in that stratum doubtful.

4. Gravidity and parity have been confused

The recorded variable is gravidity (primigravida versus multigravida) but the Abstract, Results, and Discussion repeatedly describe it as parity or multiparity. These concepts are not interchangeable. A multigravida woman may have had only pregnancy losses and no previous birth.

The interpretation that the OR of 0.114 demonstrates that women with previous vaginal-birth experience are less likely to have a cesarean is therefore unsupported. Moreover, previous cesarean is structurally nested within multigravidity: a primigravida cannot have a previous cesarean. Conditional on previous cesarean, the multigravida coefficient largely contrasts multigravidas without previous cesarean with primigravidas. This also helps explain why the crude and adjusted directions differ.

True parity and previous vaginal-delivery history should be used if available. Otherwise, the variable must be consistently called gravidity, the unsupported interpretation removed, and joint distributions of gravidity, previous cesarean, and current delivery mode presented.

5. Odds ratios are being interpreted as risks or probabilities

The manuscript describes ORs as changes in “likelihood” and states that multigravida women had an “88.6% lower likelihood” of cesarean delivery. An OR of 0.114 represents lower odds, not an 88.6% lower probability. This distinction is material because cesarean delivery occurred in 39.2% of the analytic sample.

The authors should either use odds terminology consistently or consider a modified Poisson model with robust standard errors, complemented by standardized probabilities, risk ratios, or risk differences. For context, the unadjusted cesarean proportions for session 4 were 32.3% among attendees and 45.1% among non-attendees, corresponding to an approximate risk ratio of 0.72 and risk difference of −12.7 percentage points. These are much more interpretable than the odds ratio alone.

Tables 2 and 3 should likewise show the proportion undergoing cesarean within each attendance group, rather than percentages of attendance within delivery-outcome groups.

6. Model specification and reported performance require revision

Several additional model issues should be addressed:

• Covariate inclusion is not justified using clinical knowledge or a prespecified causal framework.

• Linearity of age and gestational week on the log-odds scale is assumed without assessment. Flexible functional forms should be considered.

• “Preferred vaginal delivery” collapses cesarean preference and being undecided into a single reference category. These are conceptually different and should ordinarily be modeled separately.

• The consequences of including four potentially correlated session indicators should be assessed.

• A nonsignificant Hosmer–Lemeshow test does not demonstrate good model fit.

• Classification accuracy is of little value here and is dominated by previous cesarean history. Indeed, simply predicting cesarean for women with previous cesarean and vaginal birth for everyone else would correctly classify approximately 83.2% of participants—almost identical to the reported model accuracy of 83.8%.

Because prediction is not the study objective, classification performance and model-wide significance could be omitted in favor of well-specified effect estimation. If predictive performance is retained, discrimination, calibration, threshold choice, and internal validation would be needed.

7. Multiplicity and the exploratory nature of the fourth-session finding

Four separate session associations were examined, followed by subgroup analyses, without identification of a prespecified primary session or any multiplicity strategy. The adjusted p-value for the fourth-session coefficient is 0.011 and should not be treated as definitive evidence that its particular content is beneficial.

The authors should state whether the session-specific hypotheses were prespecified. Otherwise, the fourth-session result should be explicitly described as exploratory and hypothesis-generating. A formal interaction analysis is preferable to inferring subgroup differences from separate significance tests.

8. Missing-data reporting is incomplete

The preferred-delivery categories in Table 1 sum to 1,417 rather than 1,418, indicating at least one unreported missing value. The manuscript should give missingness for every variable, explain how missing covariates were handled, and show how the final regression sample of 641 was obtained. The RECORD checklist itself acknowledges that missingness and data-cleaning methods are not fully reported.

9. The conclusions exceed what the analysis supports

The recommendation to strengthen postpartum-related educational content is not justified by this observational session-attendance comparison. The fourth-session association could reflect who attended, when they attended, or other unmeasured clinical and behavioral factors.

At most, the current data identify an exploratory association among selected same-hospital deliverers. The Abstract, Key Messages, Discussion, and Conclusion should be revised accordingly.

Minor statistical comments

1. Clarify whether “gestational week” means gestational age at first educational attendance and explain its derivation for women attending multiple sessions.

2. Report distributions or ranges for age and gestational week and categorical frequencies for the number of sessions; mean ± SD alone is not particularly informative for a bounded, likely skewed count.

3. Provide unadjusted and adjusted effect estimates with 95% confidence intervals consistently, rather than relying heavily on p-values and chi-square statistics.

4. Explain the reversal whereby cesarean delivery was more common among multigravidas in the unadjusted analysis but the adjusted gravidity coefficient was strongly inverse.

5. If women attending the same session date or educator constitute natural clusters, assess whether cluster-robust standard errors are needed.

VERSION 1 - AUTHOR RESPONSE

Reviewer: 1<br><br>I have reviewed your study examining the relationship between prenatal education processes, birth preferences, and final delivery methods of pregnant women with great interest and satisfaction. I congratulate you on this valuable work, which has the potential to make a significant contribution to the maternal health literature, both clinically and academically.<br>The use of logistic regression modeling in analyzing multifactorial dynamics such as birth preferences and actual delivery methods has significantly elevated the level of scientific evidence in the study. The presentation of the tabular data is extremely transparent and systematically structured. The exceptional meticulousness with which all statistical details are interpreted in depth, guiding the reader without getting lost in the details, has increased the readability of the study. The findings have been rationalized within a framework of strong scientific foundations, blended with current and high-quality literature.<br>To further enhance the quality of your study and strengthen its visibility in international indexes, I am submitting a few minor points that I suggest you review in the main text, along with the attached files.	We thank the reviewer for the positive assessment of our study and for the constructive comments. We have carefully considered all suggestions and revised the manuscript accordingly.
Reducing the sentence to a few short sentences will make it easier to understand, thus improving the fluency of the article.	Thank you for this suggestion. The relevant text has been revised and simplified to improve clarity and readability.
Sharing short-term and long-term results, prioritizing those that are most valuable, can further enhance the overall picture.	Thank you. The Introduction has been revised to provide examples of important short-term maternal risks associated with cesarean section, including infection, haemorrhage, and thromboembolic complications, as well as longer-term consequences for subsequent pregnancies and child health.
Since the table presents data on the number of pregnancies, it would be appropriate to make a comment related to pregnancies here as well. If a comment regarding parity is desired, adding this data to the table would support a clearer report.	Thank you for this comment. True parity data were available and were therefore used in the revised manuscript. Parity was categorised as nullipara or multipara, added to Table 1, and used consistently in the Results, multivariable analysis, and Discussion.
In descriptive/cross-sectional studies, it is flawed to conclude the discussion by establishing cause-and-effect relationships. Rather, it is recommended that the discussion be concluded with suggestions/inferences aimed at reducing the tendency for cesarean sections in women of this age or encouraging vaginal delivery. This could include suggesting ways to lower the age of childbirth.	We agree that causal interpretation should be avoided given the retrospective observational design. The Discussion and Conclusion have therefore been revised to use association-based and cautious language, and causal interpretations have been removed.

It is recommended to include supporting literature evidence.	Thank you. Supporting literature has been added and updated in the relevant sections of the Discussion.
It would be better to update the statement as an inference sentence.	The relevant statement has been revised to present the proposed explanation as a possible interpretation rather than a definitive conclusion.
Before we go any further, it would be helpful to include the strengths and limitations of the study.	A "Strengths and limitations of this study" section has been added immediately after the Abstract. In addition, the limitations of the study are discussed in a separate paragraph in the Discussion.
Reviewer: 2<br>Comments to the Author:<br>Statistical review<br>This review is restricted to the statistical methods, analysis, reporting, and interpretation.<br>The study addresses a relevant question and includes a reasonably large initial cohort. However, several important statistical issues currently limit interpretation of the reported association between attendance at the fourth educational session and mode of delivery.	We thank the reviewer for the detailed statistical evaluation. The manuscript and analyses were substantially revised to address the concerns raised. In particular, the analytic sample and outcome availability were clarified, the primary multivariable analysis was restricted to women without a previous cesarean section, parity was corrected and used consistently, session attendance patterns and timing were described in greater detail, and the interpretation of the Session 4 finding was made explicitly exploratory.
Major comments<br>1. Selection into the analytic sample<br>Delivery outcomes were available for only 641 of 1,418 women: 45.2% of the initial cohort. Thus, 777 women (54.8%) were excluded from all outcome analyses because they delivered elsewhere or their records were unavailable. This cannot be treated as ordinary missingness without further investigation. Choice of delivery hospital may be related to educational attendance, pregnancy risk, planned cesarean delivery, socioeconomic circumstances, or other determinants of delivery mode.<br>The authors should:<br>• provide a participant flow diagram and detailed reasons for outcome non-availability;<br>• compare the 641 included women with the 777 excluded women on every available baseline and attendance variable;<br>• present Table 1 separately for the analytic sample, rather than combining characteristics of all 1,418 women with outcomes from only 641;<br>• discuss the likely direction and consequences of selection bias; and<br>• consider an inverse-probability-of-selection weighted sensitivity analysis using variables available for the complete cohort, or other methods for controlling selection bias.<br>Even after such an analysis, the target population should be described as women attending	We agree that delivery outcome availability required clearer reporting and evaluation. We have therefore clarified the follow-up status of the full cohort of 1,418 women. At the time of data extraction, 530 women had ongoing pregnancies and had not yet reached the delivery outcome window; therefore, these women were not considered to have missing delivery outcomes. Among the remaining women, delivery outcomes were available for 641, while 247 had passed their estimated delivery date but had no delivery outcome recorded in the hospital system.<br><br>A participant flow diagram has been added. Table 1 now presents the characteristics of the 641-woman analytic sample only. To assess potential selection bias, women with available delivery outcomes (n=641) were compared with women whose estimated delivery date had passed but whose outcome was unavailable (n=247) for all available baseline and education-related variables. These comparisons are presented in Supplementary Table 1.<br><br>Differences were observed in gestational age at first educational attendance, number of sessions attended, attendance at Sessions 3 and 4, parity, and previous cesarean history. These findings are now explicitly acknowledged in the Limitations

antenatal education who subsequently delivered at the same hospital, rather than all attendees.	section as evidence that selection bias cannot be excluded.<br><br>We did not perform inverse-probability-of-selection weighting because the 530 women with ongoing pregnancies had not yet experienced the outcome and therefore could not appropriately be classified together with women whose delivery outcome was unavailable. Instead, we evaluated potential selection bias among women who had reached the delivery outcome window using all available baseline and education-related variables.
2. Attendance at the fourth session cannot presently be interpreted as an effect of its content Attendance was neither randomized nor assigned according to session content. Because women could attend different and overlapping combinations of sessions, fourth-session attendance may be a marker of earlier entry into antenatal care, greater healthcare engagement, more visits, scheduling opportunity, socioeconomic characteristics, or differences in pregnancy risk. Adjustment for "gestational week at education" alone is unlikely to address this.<br>The authors should report:<br>• the distribution of the number of sessions attended in the 641-woman analytic sample;<br>• all observed attendance combinations and the correlations among the four attendance indicators;<br>• the exact scheduling and sequence of sessions;<br>• gestational age at each attended session, not merely an ambiguously defined gestational week;<br>• whether educators, calendar periods, or class groups differed; and<br>• an a priori rationale for confounder selection.<br>Clinically important confounders appear to be unavailable or omitted, including indications for cesarean delivery, fetal presentation, pregnancy complications, induction or onset of labor, previous vaginal birth, planned cesarean delivery, antenatal-care utilization, and relevant socioeconomic factors. Without adequate control for these variables, the analysis cannot support conclusions about the distinctive value of postpartum-focused content.	We agree and have substantially expanded the description of the antenatal education programme and attendance pattern.<br><br>The revised manuscript now reports:<br><br>the distribution of the number of sessions attended in the analytic sample;<br>all observed attendance combinations;<br>pairwise associations among the four session-attendance indicators using Phi coefficients;<br>the gestational timing of attendance at each individual session;<br>the sequence and content of all four sessions; and the organisation of the education programme.<br><br>All sessions were delivered by the same educator at a fixed time each day in the outpatient waiting area. The programme structure and session content remained consistent throughout the study period. No predefined class groups were formed; participation depended on which pregnant women were present in the outpatient waiting area at the scheduled education time.<br><br>The primary multivariable model included maternal age, parity, gestational week at first educational attendance, pre-education preferred mode of delivery, and attendance at Sessions 1–4. These variables were selected based on their clinical relevance to mode of delivery, the study objectives, and their availability in the routinely collected dataset.<br><br>We also agree that several clinically important potential confounders were unavailable as comparable pre-delivery variables for the full analytic sample. Cesarean indications were available and are now presented descriptively in Supplementary Table 5. However, because these indications represent outcome-related or intrapartum information and were not

	comparable pre-delivery covariates for all women, they were not included in the regression model.<br><br>The Discussion and Conclusion have therefore been revised to avoid attributing the observed association specifically to the content of Session 4. The finding is now explicitly described as exploratory and may reflect both the content and timing of Session 4, as well as characteristics of women who attended later in pregnancy.
3. Quasi-complete separation is acknowledged but not addressed analytically<br>From Tables 1 and 3, it can be inferred that 145 of 147 women with a previous cesarean underwent another cesarean, while only two delivered vaginally. Consequently, the maximum-likelihood estimate for previous cesarean is severely affected by quasi-complete separation, producing the unstable OR of 821.4.<br>Simply acknowledging this limitation in the Discussion does not resolve the problem. The authors should either:<br>• make the adjusted analysis among women without a previous cesarean the primary analysis for the educational-session question; or<br>• refit the full model using a separation-robust method, such as Firth penalized logistic regression or an appropriately specified Bayesian model.<br>Table 3 contains only unadjusted chi-square analyses. An adjusted model among the 494 women without previous cesarean is essential. An interaction between previous cesarean and session attendance could theoretically be examined, although only two vaginal births among women with previous cesarean make meaningful estimation in that stratum doubtful.	We agree. The previous full-sample logistic regression model was affected by quasi-complete separation because almost all women with a previous cesarean section underwent repeat cesarean delivery.<br><br>To address this problem, the primary multivariable analysis was redefined and restricted to women without a history of previous cesarean section (n=494). A new adjusted logistic regression model was performed in this subgroup.<br><br>Previous cesarean history was therefore no longer included as a predictor in the primary model. The revised model evaluates maternal age, parity, gestational week at first educational attendance, pre-education preferred mode of delivery, and attendance at Sessions 1–4. The adjusted results are now presented in Table 4.<br><br>This approach avoids the unstable estimate produced by quasi-complete separation and provides a more clinically interpretable assessment of the association between antenatal education attendance and mode of delivery.
4. Gravidity and parity have been confused<br>The recorded variable is gravidity (primigravida versus multigravida) but the Abstract, Results, and Discussion repeatedly describe it as parity or multiparity. These concepts are not interchangeable. A multigravida woman may have had only pregnancy losses and no previous birth.<br>The interpretation that the OR of 0.114 demonstrates that women with previous vaginal-birth experience are less likely to have a cesarean is therefore unsupported. Moreover, previous cesarean is structurally nested within multigravidity: a primigravida cannot have a previous cesarean. Conditional on previous cesarean, the multigravida coefficient largely	Thank you for identifying this important issue. We rechecked the source data and confirmed that the available variable represents parity rather than gravidity.<br><br>The terminology has therefore been corrected throughout the manuscript. Participants are now classified as nulliparous or multiparous, parity is presented in Table 1, and parity is used consistently in the Abstract, Methods, Results, regression analysis, and Discussion.<br><br>The interpretation suggesting that the original gravidity variable represented previous vaginal-birth experience has also been removed.

contrasts multigravidas without previous cesarean with primigravidas. This also helps explain why the crude and adjusted directions differ.<br>True parity and previous vaginal-delivery history should be used if available. Otherwise, the variable must be consistently called gravidity, the unsupported interpretation removed, and joint distributions of gravidity, previous cesarean, and current delivery mode presented.	In addition, because previous cesarean history strongly influenced the crude relationship between parity and current mode of delivery, the primary adjusted analysis was restricted to women without a previous cesarean section. The Discussion now explains why the crude cesarean rate was higher among multiparous women in the overall sample, whereas multiparity was associated with lower adjusted odds of cesarean delivery in the restricted primary analysis.
5. Odds ratios are being interpreted as risks or probabilities<br>The manuscript describes ORs as changes in “likelihood” and states that multigravida women had an “88.6% lower likelihood” of cesarean delivery. An OR of 0.114 represents lower odds, not an 88.6% lower probability. This distinction is material because cesarean delivery occurred in 39.2% of the analytic sample.<br>The authors should either use odds terminology consistently or consider a modified Poisson model with robust standard errors, complemented by standardized probabilities, risk ratios, or risk differences. For context, the unadjusted cesarean proportions for session 4 were 32.3% among attendees and 45.1% among non-attendees, corresponding to an approximate risk ratio of 0.72 and risk difference of −12.7 percentage points. These are much more interpretable than the odds ratio alone.<br>Tables 2 and 3 should likewise show the proportion undergoing cesarean within each attendance group, rather than percentages of attendance within delivery-outcome groups.	We agree. The manuscript has been revised to use odds terminology consistently when interpreting logistic regression results.<br><br>Statements referring to percentage reductions in “likelihood” or probability based directly on odds ratios have been removed. Adjusted odds ratios with 95% confidence intervals are now reported and interpreted as higher or lower odds of cesarean delivery.<br><br>Tables 2 and 3 have also been reformatted so that percentages represent the proportion of vaginal and cesarean deliveries within each session-attendance group. This provides clinically interpretable unadjusted proportions in addition to the adjusted odds ratios from the regression analysis.
6. Model specification and reported performance require revision<br>Several additional model issues should be addressed:<br>• Covariate inclusion is not justified using clinical knowledge or a prespecified causal framework.<br>• Linearity of age and gestational week on the log-odds scale is assumed without assessment. Flexible functional forms should be considered.<br>• “Preferred vaginal delivery” collapses cesarean preference and being undecided into a single reference category. These are conceptually different and should ordinarily be modeled separately.<br>• The consequences of including four potentially correlated session indicators should be assessed.<br>• A nonsignificant Hosmer–Lemeshow test does not demonstrate good model fit.	The model specification and reporting have been revised as suggested.<br><br>Maternal age, parity, gestational week at first educational attendance, pre-education preferred mode of delivery, and attendance at Sessions 1–4 were included in the primary model based on clinical relevance, the study objectives, and the variables available in the routinely collected dataset.<br><br>Linearity of maternal age and gestational week at first educational attendance on the logit scale was assessed using the Box–Tidwell approach. The interaction terms with their natural logarithms were not statistically significant (age: p=0.148; gestational week: p=0.208), indicating no evidence of violation of the linearity assumption.

• Classification accuracy is of little value here and is dominated by previous cesarean history. Indeed, simply predicting cesarean for women with previous cesarean and vaginal birth for everyone else would correctly classify approximately 83.2% of participants—almost identical to the reported model accuracy of 83.8%.<br>Because prediction is not the study objective, classification performance and model-wide significance could be omitted in favor of well-specified effect estimation. If predictive performance is retained, discrimination, calibration, threshold choice, and internal validation would be needed.	Pre-education preferred mode of delivery is now modelled using three categories: preferred vaginal delivery, preferred cesarean delivery, and undecided, rather than combining cesarean preference and undecided participants.<br><br>Pairwise associations among the four session-attendance indicators were examined and are reported using Phi coefficients in Supplementary Table 3.<br><br>The interpretation of the Hosmer–Lemeshow test as demonstrating "good fit" has been removed. The test statistic and p-value are now reported descriptively together with Nagelkerke R^2.<br><br>Classification accuracy has also been removed because prediction was not the objective of the study.
7. Multiplicity and the exploratory nature of the fourth-session finding<br>Four separate session associations were examined, followed by subgroup analyses, without identification of a prespecified primary session or any multiplicity strategy. The adjusted p-value for the fourth-session coefficient is 0.011 and should not be treated as definitive evidence that its particular content is beneficial.<br>The authors should state whether the session-specific hypotheses were prespecified. Otherwise, the fourth-session result should be explicitly described as exploratory and hypothesis-generating. A formal interaction analysis is preferable to inferring subgroup differences from separate significance tests.	We agree. No individual session had been specified a priori as the primary educational exposure, and four session-specific associations were examined without formal multiplicity adjustment.<br><br>The manuscript has therefore been revised to explicitly describe the association observed for Session 4 as exploratory and hypothesis-generating.<br><br>This qualification is now included in the Abstract, Discussion, Limitations, and interpretation of the study findings. We also state that the observed association requires confirmation in prospective studies and should not be interpreted as evidence that the specific content of Session 4 causes a reduction in cesarean delivery.
8. Missing-data reporting is incomplete<br>The preferred-delivery categories in Table 1 sum to 1,417 rather than 1,418, indicating at least one unreported missing value. The manuscript should give missingness for every variable, explain how missing covariates were handled, and show how the final regression sample of 641 was obtained. The RECORD checklist itself acknowledges that missingness and data-cleaning methods are not fully reported.	The reporting of outcome availability and analytic sample derivation has been revised.<br><br>The full cohort consisted of 1,418 women. At data extraction, 530 had ongoing pregnancies and had not yet reached the delivery outcome window. Delivery outcomes were available for 641 women, while 247 women had passed their estimated delivery date but had no delivery outcome available in the hospital records.<br><br>The 641 women with available delivery outcomes constituted the analytic sample for mode-of-delivery analyses. The primary regression analysis was further restricted to the

	494 women without a previous cesarean section because of quasi-complete separation.<br><br>Table 1 has been reconstructed using the analytic sample only, and the participant flow is now shown in a flow diagram.
9. The conclusions exceed what the analysis supports<br>The recommendation to strengthen postpartum-related educational content is not justified by this observational session-attendance comparison. The fourth-session association could reflect who attended, when they attended, or other unmeasured clinical and behavioral factors.<br>At most, the current data identify an exploratory association among selected same-hospital deliverers. The Abstract, Key Messages, Discussion, and Conclusion should be revised accordingly.	We agree and have revised the Abstract, Discussion, and Conclusion to remove causal or prescriptive statements regarding the fourth educational session.<br><br>The previous recommendation that strengthening postpartum-related education might increase vaginal delivery rates has been removed.<br><br>The revised manuscript now states that attendance at Session 4 was associated with mode of delivery but that this association may reflect the timing of attendance, characteristics of the women attending the session, and other unmeasured factors. The finding is explicitly described as exploratory and hypothesis-generating and is proposed for further evaluation in prospective studies.
Minor statistical comments<br>1. Clarify whether “gestational week” means gestational age at first educational attendance and explain its derivation for women attending multiple sessions.	This has been clarified throughout the manuscript as “gestational week at first educational attendance.” In addition, gestational age at attendance for each individual session is now reported separately in Supplementary Table 4.
2. Report distributions or ranges for age and gestational week and categorical frequencies for the number of sessions; mean ± SD alone is not particularly informative for a bounded, likely skewed count.	The descriptive reporting has been expanded. Gestational week at first educational attendance and number of sessions attended are now presented using medians and interquartile ranges where appropriate. The frequencies of women attending one, two, three, or four sessions are also reported. Gestational timing and minimum–maximum ranges for each individual session are presented in Supplementary Table 4.
3. Provide unadjusted and adjusted effect estimates with 95% confidence intervals consistently, rather than relying heavily on p-values and chi-square statistics.	Unadjusted delivery proportions are now presented clearly in Tables 2 and 3, and adjusted odds ratios with 95% confidence intervals are reported consistently in the primary multivariable model in Table 4.
4. Explain the reversal whereby cesarean delivery was more common among multigravidas in the unadjusted analysis but the adjusted gravidity coefficient was strongly inverse.	This issue has been addressed following correction of the variable from gravidity to parity. In the overall analytic sample, the crude cesarean rate was higher among multiparous women. However, previous cesarean history was considerably more common among multiparous women and strongly influenced current mode of delivery.

	The primary multivariable analysis was therefore restricted to women without a previous cesarean section. In this clinically more homogeneous group, multiparity was associated with lower adjusted odds of cesarean delivery. This apparent reversal and its likely explanation are now explicitly discussed in the manuscript.
5. If women attending the same session date or educator constitute natural clusters, assess whether cluster-robust standard errors are needed.	We have clarified the organisation of the education programme in the Methods section. All sessions were delivered by the same educator at a fixed time each day in the outpatient waiting area, and the programme structure and session content remained consistent throughout the study period. No predefined class groups were formed; attendance depended on which pregnant women were present in the outpatient waiting area at the scheduled education time.<br><br>Because there were no distinct educators or predefined class groups, educator-level or class-level clustering was not present as a structured feature of the programme. Individual daily session identifiers were not available in the retrospective dataset; therefore, clustering by individual session date could not be formally evaluated.

VERSION 2 - REVIEW

Reviewer 2

Name Lamadrid-Figueroa, Hector

Affiliation National Institute of Public Health, Department of Perinatal Health

Date 30-Aug-2026

COI

Thank you for the careful and substantial revision. The manuscript has improved considerably. In particular, the authors have appropriately restricted the primary multivariable analysis to women without a previous cesarean section, corrected the gravidity/parity terminology, improved the presentation of the analytic sample and attendance patterns, reported session-specific timing, and moderated several causal interpretations.

Nevertheless, two important statistical issues remain unresolved. One arises from the authors' response to the previous review, while the other has become apparent from the newly reported gestational timing of the sessions.

1. Selection into the analytic sample and the proposed weighting analysis

The reason given for not conducting inverse-probability-of-selection weighting appears to reflect a misunderstanding of the proposed analysis. I agree that the 530 women whose pregnancies were ongoing at data extraction should not be classified as having missing delivery outcomes. These women need not be included in the selection analysis.

The weighting analysis can instead be conducted among the 888 women who had reached the delivery-outcome window: 641 women with an available delivery outcome and 247 women whose estimated delivery date had passed but whose delivery outcome was unavailable.

Within this group, the probability of outcome availability can be modelled using the baseline, obstetric, and attendance variables available for all participants. Stabilized inverse-probability weights can then be applied to the women with observed outcomes when repeating the primary analysis. Because the primary outcome model is restricted to women without a previous cesarean section, the authors could alternatively estimate the selection model directly within that target population: 494 women with observed outcomes and 217 without an available outcome.

This sensitivity analysis is particularly important because Supplementary Table 1 does not reassure against selection bias; it demonstrates that outcome availability was associated with the principal exposure and other relevant variables. Session 4 attendance was 46.3% among women with observed outcomes versus 31.6% among women without an available outcome. Differences were also observed in Session 3 attendance, number of sessions, parity, and previous cesarean history. A comparison of characteristics and an acknowledgment in the Limitations section do not quantify or correct the resulting selection.

The authors should also report the exact data-extraction date. Enrollment ended in July 2025, whereas this revised manuscript was submitted in August 2026. All pregnancies in the source cohort should therefore now have reached completion. If permitted and technically feasible, updating delivery-outcome ascertainment would be preferable to retaining an administratively immature data snapshot. If follow-up cannot be updated, the reason should be explained.

2. Late-pregnancy opportunity and guarantee-time bias affecting Session 4

Supplementary Table 4 shows that the sessions occurred at markedly different gestational ages: median 14 weeks for Session 1, 22 weeks for Session 2, 28 weeks for Session 3, and 36 weeks for Session 4.

Consequently, attendance at Session 4 ordinarily required a woman to remain pregnant until late gestation. The non-attendance group may include women who delivered before they

had an opportunity to attend Session 4. Earlier or complicated deliveries may also be more likely to involve cesarean section. This creates an opportunity or guarantee-time bias that could itself generate an apparent inverse association between Session 4 attendance and cesarean delivery.

Adjustment for gestational week at first educational attendance does not address this mechanism.

The description stating that sessions were delivered by the same educator at a fixed time does not fully explain how women became eligible or were selected for the different sessions. The authors should clarify whether the four topics were offered according to a fixed calendar rotation or selected according to the woman's gestational age, whether all women had an opportunity to attend Session 4 before delivery, the gestational-age window in which Session 4 was ordinarily offered, gestational age at delivery, and how many women classified as Session 4 non-attenders delivered before the usual Session 4 window.

If the necessary dates are available, the Session 4 analysis should be repeated using an appropriately defined landmark, risk-set, or time-varying approach based on actual eligibility and attendance timing. Women who had already delivered before having an opportunity to attend cannot serve as an unexposed comparison group for estimating the association with Session 4 attendance.

If the available data cannot address this problem, the Session 4 result is not sufficiently interpretable to remain the manuscript's central substantive finding.

3. Interpretation of the Session 4 association

The revised manuscript appropriately describes the finding as exploratory in several places. However, the Discussion still states that Session 4 may have "distinctive relevance," and the Conclusion suggests that its "timing and content" may be relevant to delivery outcomes.

The analysis cannot distinguish the session's content from its late timing, the clinical characteristics of women who remained pregnant and attended at that time, or other determinants of attendance. These statements should therefore be removed or narrowed substantially. At most, the current analysis identifies an exploratory association requiring confirmation after accounting for eligibility, timing, outcome ascertainment, and clinical confounding.

4. RECORD checklist

The RECORD checklist does not appear to have been updated to correspond to the revised manuscript. For example, it still states that no flow diagram was included, refers to gravidity and classification accuracy, says that no separate Limitations section is present, and identifies the funding statement as unreported. These statements now contradict the manuscript. The complete checklist should be reviewed and corrected before publication.

5. Minor comment

The Strengths and limitations section describes the covariates as "prespecified," but the Methods and response letter state that they were selected based on clinical relevance, the study objectives, and availability in the routinely collected dataset. Unless they were documented prospectively in a protocol or statistical analysis plan, "prespecified" should be removed.

The authors have satisfactorily addressed most of the original statistical concerns, and the revision represents a genuine improvement. However, selective outcome ascertainment and the late-pregnancy opportunity bias remain central to interpretation of the Session 4 association. I therefore recommend another focused major revision. If the timing bias cannot be addressed analytically, the manuscript will need to be reframed so that the Session 4 result is no longer presented as its principal finding.

VERSION 2 - AUTHOR RESPONSE

Reviewer: 2<br>Comments to the Author:	Responds
Thank you for the careful and substantial revision. The manuscript has improved considerably. In particular, the authors have appropriately restricted the primary multivariable analysis to women without a previous cesarean section, corrected the gravidity/parity terminology, improved the presentation of the analytic sample and attendance patterns, reported session-specific timing, and moderated several causal interpretations.<br><br>Nevertheless, two important statistical issues remain unresolved. One arises from the authors' response to the previous review, while the other has become apparent from the newly reported gestational timing of the sessions.	Thank you for this careful assessment. We appreciate the reviewer's acknowledgement of the improvements made in the revised manuscript. We have addressed the two remaining statistical concerns in the current revision by conducting an inverse-probability-of-selection weighted sensitivity analysis for selective outcome ascertainment and by substantially reframing the Session 4 finding to account for the possibility of late-pregnancy opportunity bias. The relevant Methods, Results, Discussion, and Conclusion sections have been revised accordingly.
1. Selection into the analytic sample and the proposed weighting analysis<br><br>The reason given for not conducting inverse-probability-of-selection weighting appears to reflect a misunderstanding of the proposed analysis. I agree that the 530 women whose pregnancies were ongoing at data extraction should not be classified as having missing delivery outcomes. These women need not be included in the selection analysis.	Thank you for this important clarification. We agree that the 530 women whose pregnancies were still ongoing at the time of data extraction should not be included in the assessment of selection related to delivery-outcome availability. We have therefore revised our approach accordingly.<br>First, we have clarified the timing of data extraction in the Methods. The data were extracted at 31 July 2025. At that time, 530 women had ongoing pregnancies and had not yet reached the delivery-outcome window; therefore, they were not treated as having missing delivery

The weighting analysis can instead be conducted among the 888 women who had reached the delivery-outcome window: 641 women with an available delivery outcome and 247 women whose estimated delivery date had passed but whose delivery outcome was unavailable.<br><br>Within this group, the probability of outcome availability can be modelled using the baseline, obstetric, and attendance variables available for all participants. Stabilized inverse-probability weights can then be applied to the women with observed outcomes when repeating the primary analysis. Because the primary outcome model is restricted to women without a previous cesarean section, the authors could alternatively estimate the selection model directly within that target population: 494 women with observed outcomes and 217 without an available outcome.<br><br>This sensitivity analysis is particularly important because Supplementary Table 1 does not reassure against selection bias; it demonstrates that outcome availability was associated with the principal exposure and other relevant variables. Session 4 attendance was 46.3% among women with observed outcomes versus 31.6% among women without an available outcome. Differences were also observed in Session 3 attendance, number of sessions, parity, and previous cesarean history. A comparison of characteristics and an acknowledgment in the Limitations section do not quantify or correct the resulting selection.<br><br>The authors should also report the exact data-extraction date. Enrollment ended in July 2025, whereas this revised manuscript was submitted in August 2026. All pregnancies in the source cohort should therefore now have reached completion. If permitted and technically feasible, updating delivery-outcome ascertainment would be preferable to retaining an administratively immature data snapshot. If follow-up cannot be updated, the reason should be explained.	outcomes. Among women who had reached the delivery-outcome window, delivery outcomes were available for 641 women and unavailable for 247 women. This distinction is now explicitly stated in the manuscript.<br>Second, following the reviewer's recommendation, we performed an inverse-probability-of-selection weighted sensitivity analysis restricted to the population corresponding to our primary analysis, namely women without a previous cesarean section who had reached the delivery-outcome window. This target population comprised 711 women: 494 with observed delivery outcomes and 217 with unavailable delivery outcomes. The probability of having an observed delivery outcome was estimated using logistic regression including maternal age, parity, gestational week at first educational attendance, pre-education preferred mode of delivery, and attendance at Sessions 1–4. Stabilized inverse-probability-of-selection weights were then calculated and applied to the 494 women with observed outcomes, and the primary multivariable logistic regression model was repeated as a weighted sensitivity analysis. These procedures have now been added to the Data Analysis section.<br>The use of inverse probability weighting to address potential selection bias arising from non-random outcome availability is supported by methodological literature. Inverse probability weighting reweights individuals with observed outcomes according to their probability of being selected into the analytic sample, thereby creating a pseudo-population that more closely represents the target population under the assumptions of the selection model (Carry et al., 2021). Stabilized weighting has also been applied to account for attrition and non-response in longitudinal pregnancy cohorts and can reduce variability associated with unstabilized weights (Developing non-response weights to account for attrition-related bias in a longitudinal pregnancy cohort, 2023).<br>The weighted results were broadly consistent with the primary analysis. Maternal age remained associated with higher odds of cesarean delivery (weighted aOR 1.097, 95% CI 1.041–1.156; $p<0.001$), whereas multiparity remained associated with lower odds of cesarean delivery (weighted aOR 0.139, 95% CI 0.076–0.255; $p<0.001$). Attendance at Session 4 also remained associated with lower odds of cesarean delivery (weighted aOR 0.510, 95% CI 0.285–0.913; $p=0.023$). Gestational week at first educational

	attendance, pre-education preferred mode of delivery, and attendance at Sessions 1–3 remained non-significant. The complete comparison between the primary and weighted analyses is now presented in Supplementary Table 6.<br>We have also revised the Limitations section to acknowledge that, although the weighted sensitivity analysis yielded estimates broadly consistent with the primary analysis, residual selection bias related to unmeasured factors cannot be excluded.<br>Finally, we agree that updating delivery-outcome ascertainment would have been preferable if permitted. However, additional follow-up could not be undertaken because the ethical and institutional permissions covered the original data-extraction period only and did not permit subsequent collection of additional outcome data. We have therefore clarified the original extraction timing and addressed potential selection due to unavailable outcomes analytically through the inverse-probability-of-selection weighted sensitivity analysis.<br>**References:**<br>Carry PM, Vanderlinden LA, Dong F, et al. Inverse probability weighting is an effective method to address selection bias during the analysis of high dimensional data. *Genet Epidemiol.* 2021;45(6):593–603. doi:10.1002/gepi.22418.<br>Pitt TM, Hetherington E, Adhikari K, Premji S, Racine N, Tough SC, McDonald S. Developing non-response weights to account for attrition-related bias in a longitudinal pregnancy cohort. BMC Med Res Methodol. 2023 Dec 14;23(1):295. doi: 10.1186/s12874-023-02121-1. PMID: 38097944; PMCID: PMC10722744.
2. Late-pregnancy opportunity and guarantee-time bias affecting Session 4<br><br>Supplementary Table 4 shows that the sessions occurred at markedly different gestational ages: median 14 weeks for Session 1, 22 weeks for Session 2, 28 weeks for Session 3, and 36 weeks for Session 4.<br><br>Consequently, attendance at Session 4 ordinarily required a woman to remain pregnant until late gestation. The non-attendance group may include women who delivered before they had an opportunity to attend Session 4. Earlier or complicated deliveries may also be more likely to involve cesarean section. This creates an opportunity or guarantee-time bias that could	Thank you for highlighting this important issue. We agree that the later observed timing of Session 4 creates the possibility of opportunity, or guarantee-time, bias and that this requires clearer description and more cautious interpretation.<br><br>We have therefore clarified the structure of the antenatal education programme in the Methods. The programme comprised four predefined session topics, but sessions were not assigned according to gestational age and did not follow a fixed calendar rotation. At the scheduled education time, pregnant women present in the outpatient waiting area were offered the available session topics, and the topic delivered that day was selected according to the preferences of the

itself generate an apparent inverse association between Session 4 attendance and cesarean delivery.<br><br>Adjustment for gestational week at first educational attendance does not address this mechanism.<br><br>The description stating that sessions were delivered by the same educator at a fixed time does not fully explain how women became eligible or were selected for the different sessions. The authors should clarify whether the four topics were offered according to a fixed calendar rotation or selected according to the woman's gestational age, whether all women had an opportunity to attend Session 4 before delivery, the gestational-age window in which Session 4 was ordinarily offered, gestational age at delivery, and how many women classified as Session 4 non-attenders delivered before the usual Session 4 window.<br><br>If the necessary dates are available, the Session 4 analysis should be repeated using an appropriately defined landmark, risk-set, or time-varying approach based on actual eligibility and attendance timing. Women who had already delivered before having an opportunity to attend cannot serve as an unexposed comparison group for estimating the association with Session 4 attendance.<br><br>If the available data cannot address this problem, the Session 4 result is not sufficiently interpretable to remain the manuscript's central substantive finding.	women present. Importantly, there were no predefined gestational-age eligibility windows for any of the four sessions. Thus, the observed gestational timing of attendance reflects actual attendance patterns rather than programme-defined gestational-age requirements.<br><br>Nevertheless, we agree that this does not eliminate the possibility of differential opportunity to attend Session 4. In the observed data, Session 4 was attended later in pregnancy, with a median gestational age of 36 weeks. Gestational age at delivery was not available in the routinely collected dataset. Therefore, we were unable to determine how many women classified as non-attenders had delivered before having an opportunity to attend Session 4. For the same reason, a landmark, risk-set, or time-varying analysis based on eligibility for Session 4 could not be performed.<br><br>In response to this concern, we have substantially revised the Discussion and narrowed the interpretation of the Session 4 finding. We no longer attribute the observed association to the specific content of Session 4. The revised text now explicitly states that the association may reflect differences in opportunity for attendance, characteristics of women who remained pregnant and continued attending antenatal care later in gestation, or other unmeasured clinical factors. The finding is therefore described as exploratory and hypothesis-generating, requiring confirmation in prospective studies that explicitly account for eligibility and timing of attendance.<br><br>We believe these revisions more accurately reflect what can and cannot be inferred from the available retrospective data and directly acknowledge the potential for late-pregnancy opportunity bias.
3. Interpretation of the Session 4 association<br><br>The revised manuscript appropriately describes the finding as exploratory in several places. However, the Discussion still states that Session 4 may have "distinctive relevance," and the Conclusion suggests that its "timing and content" may be relevant to delivery outcomes.<br><br>The analysis cannot distinguish the session's content from its late timing, the clinical characteristics of women who remained pregnant and attended at that time, or other determinants	We agree that the previous interpretation placed too much emphasis on the potential relevance of the content and timing of Session 4, although the available data do not allow these explanations to be distinguished from differences in opportunity for attendance or other clinical characteristics.<br><br>We have therefore substantially narrowed the interpretation of this finding. In the revised Discussion, we removed the suggestion that Session 4 has a "distinctive relevance" for delivery outcomes and deleted the proposed content-specific mechanism linking postpartum

of attendance. These statements should therefore be removed or narrowed substantially. At most, the current analysis identifies an exploratory association requiring confirmation after accounting for eligibility, timing, outcome ascertainment, and clinical confounding.	education to mode of delivery. The revised Discussion now explicitly states that the observed association may reflect differences in opportunity for attendance, characteristics of women who remained pregnant and continued attending antenatal care later in gestation, or other unmeasured clinical factors, rather than an effect attributable to the content of Session 4 itself. The finding is now described as exploratory and hypothesis-generating.<br><br>We have also revised the Conclusion to avoid implying that either the timing or content of Session 4 explains the observed association. The conclusion now emphasizes maternal age and parity as the main independently associated factors and presents the Session 4 association separately as an exploratory finding requiring confirmation in prospective studies that explicitly account for eligibility and timing of attendance.<br><br>We believe these revisions more appropriately reflect the observational design and the limitations of the available data.
4. RECORD checklist<br><br>The RECORD checklist does not appear to have been updated to correspond to the revised manuscript. For example, it still states that no flow diagram was included, refers to gravidity and classification accuracy, says that no separate Limitations section is present, and identifies the funding statement as unreported. These statements now contradict the manuscript. The complete checklist should be reviewed and corrected before publication.	We agree. The RECORD checklist had not been fully updated to reflect the revised manuscript. We have now reviewed and corrected the checklist throughout, including the entries on the participant flow diagram, parity, model assessment, sensitivity analysis, Limitations section, funding, generalisability, and data availability. The revised checklist has been resubmitted.
5. Minor comment<br><br>The Strengths and limitations section describes the covariates as "prespecified," but the Methods and response letter state that they were selected based on clinical relevance, the study objectives, and availability in the routinely collected dataset. Unless they were documented prospectively in a protocol or statistical analysis plan, "prespecified" should be removed.<br><br>The authors have satisfactorily addressed most of the original statistical concerns, and the revision represents a genuine improvement. However, selective outcome ascertainment and the late-pregnancy opportunity bias remain central to	We agree with the reviewer. The term "prespecified" was not appropriate because the covariates were not prospectively documented in a protocol or statistical analysis plan. We have therefore removed this term from the Strengths and limitations section and revised the text to reflect the actual covariate-selection approach described in the Methods. The revised statement now indicates that the model included maternal, obstetric, and education-related covariates selected based on clinical relevance, the study objectives, and their availability in the routinely collected dataset.<br><br>We also carefully considered the reviewer's overall recommendation that the Session 4

interpretation of the Session 4 association. I therefore recommend another focused major revision. If the timing bias cannot be addressed analytically, the manuscript will need to be reframed so that the Session 4 result is no longer presented as its principal finding.	finding should no longer be presented as the principal finding if the late-pregnancy timing bias could not be addressed analytically. Because gestational age at delivery was not available, a landmark, risk-set, or time-varying analysis could not be performed. We therefore reframed the manuscript accordingly. The Abstract now presents maternal age and parity as the main independently associated factors and describes the Session 4 association explicitly as exploratory.<br><br>The Discussion has also been revised to emphasize that the Session 4 association may reflect differences in opportunity for attendance, characteristics of women who remained pregnant and continued attending antenatal care later in gestation, or other unmeasured clinical factors, rather than an effect attributable to the content of Session 4 itself. The finding is now described as exploratory and hypothesis-generating.<br><br>Finally, the Conclusion has been revised so that maternal age and parity are presented as the principal findings, while the Session 4 association is presented separately as an exploratory observation requiring confirmation in prospective studies that explicitly account for eligibility and timing of attendance.<br><br>We believe these revisions more appropriately reflect the limitations of the retrospective design and address the reviewer's concern regarding the interpretation and prominence of the Session 4 finding.

VERSION 3 - REVIEW

Reviewer 2

Name Lamadrid-Figueroa, Hector

Affiliation National Institute of Public Health, Department of Perinatal Health

Date 07-Oct-2026

COI

The authors have satisfactorily addressed my statistical and interpretative concerns.
